# Multiple Survival Outcome Prediction of Glioblastoma Patients Based on Multiparametric MRI

**DOI:** 10.3389/fonc.2021.778627

**Published:** 2021-11-25

**Authors:** Bin Wang, Shan Zhang, Xubin Wu, Ying Li, Yueming Yan, Lili Liu, Jie Xiang, Dandan Li, Ting Yan

**Affiliations:** ^1^ College of Information and Computer, Taiyuan University of Technology, Taiyuan, China; ^2^ Department of Pathology & Shanxi Translational Medicine Research Center on Esophageal Cancer, Shanxi Medical University, Taiyuan, China

**Keywords:** multiparametric MRI, multi-survival indicators, glioblastoma, machine learning, radiomics analysis

## Abstract

**Purpose:**

Construction of radiomics models for the individualized estimation of multiple survival stratification in glioblastoma (GBM) patients using the multiregional information extracted from multiparametric MRI that could facilitate clinical decision-making for GBM patients.

**Materials and Methods:**

A total of 134 eligible GBM patients were selected from The Cancer Genome Atlas. These patients were separated into the long-term and short-term survival groups according to the median of individual survival indicators: overall survival (OS), progression-free survival (PFS), and disease-specific survival (DSS). Then, the patients were divided into a training set and a validation set in a ratio of 2:1. Radiomics features (n = 5,152) were extracted from multiple regions of the GBM using multiparametric MRI. Then, radiomics signatures that are related to the three survival indicators were respectively constructed using the analysis of variance (ANOVA) and the least absolute shrinkage and selection operator (LASSO) regression for each patient in the training set. Based on a Cox proportional hazards model, the radiomics model was further constructed by combining the signature and clinical risk factors.

**Results:**

The constructed radiomics model showed a promising discrimination ability to differentiate in the training set and validation set of GBM patients with survival indicators of OS, PFS, and DSS. Both the four MRI modalities and five tumor subregions have different effects on the three survival indicators of GBM. The favorable calibration and decision curve analysis indicated the clinical decision value of the radiomics model. The performance of models of the three survival indicators was different but excellent; the best model achieved C indexes of 0.725, 0.677, and 0.724, respectively, in the validation set.

**Conclusion:**

Our results show that the proposed radiomics models have favorable predictive accuracy on three survival indicators and can provide individualized probabilities of survival stratification for GBM patients by using multiparametric and multiregional MRI features.

## Introduction

Glioblastoma (GBM) is the most common primary malignant neoplasm in adults and is nearly uniformly fatal (1), with a median survival time about 12–14 months (2). It is necessary to establish a survival prediction model that is helpful to the treatment decision-making and disease management for GBM patients (3). In clinical studies for GBM, 5-year or 10-year benchmark survival rates are often calculated to convey prognostic information. A recent study pointed out that three survival endpoints including overall survival (OS), progression-free survival (PFS), and disease-specific survival (DSS) can be used in the study of GBM with confidence (4). It is very important to have a sufficiently long follow-up time to capture the events of interest, and the minimum follow-up time needed depends on both the aggressiveness of the type of endpoint (5).

As a noninvasive and preoperative routine examination for GBM (6), the magnetic resonance imaging (MRI) can comprehensively and macroscopically display the whole tumor and provide fine tumor features, including tumor location, shape, size, and heterogeneity (7). Currently, MRI techniques have great potential for predicting the survival of GBM patients (6, 8, 9). More recently, the field of radiomics has been introduced to extract high-throughput quantitative imaging features from MRI, transform the features into minable data, and establish a prediction or prognosis model connecting image features and tumor phenotype (10, 11). In common MRI acquisitions, four image sequences, i.e., T1-weighted gadolinium contrast-enhanced (T1CE), T1-weighted (T1), T2-weighted (T2), and T2-weighted fluid-attenuated inversion recovery (FLAIR) sequences, are recommended for the diagnosis of a brain tumor (12). It is widely believed that multiparametric MRI can improve the diagnostic efficiency and performance of survival stratification (13). Moreover, the heterogeneity of GBM is reflected in the fact that it usually contains different heterogeneous subregions (such as edema, enhanced and non-enhanced core); this inherent heterogeneity is also reflected in its imaging phenotype because its subregions are described by different intensity distributions of multimodal MRI scanning, reflecting the differences in tumor biology, which all contribute to prognosis prediction (8, 9, 14). Although previous studies have explored the survival time of GBM patients, most of them focus on OS, and the research on the heterogeneity within the tumor is insufficient. Therefore, it is necessary and feasible that a multiparametric MRI- and multiregion-based radiomics approach may improve the performance of the multi-survival stratification in GBM patients.

The aim of the present study was to develop and validate radiomics models based on multiparameter and multiregion MRI for the individualized estimation of the multiple survival indicator stratification in GBM patients.

## Materials and Methods

### Patient Population and Study Design

A total of 134 patients (i) with clinical information (such as OS of patients) from The Cancer Genome Atlas (TCGA^1^) GBM Project and (ii) the corresponding MRI data from The Cancer Imaging Archive (TCIA^2^) were retrospectively included, with the following criteria, (i) including all four types of MRI sequences (T1C, T1, T2, FLAIR); (ii) the MRI sequences were acquired prior to surgery or biopsy; (iii) the MRI sequences were acquired without excessive movement or artifacts; (iv) sufficient clinical data on OS, PFS, or DSS. Additional clinical data for all patients, including age, gender, race, and Karnofsky Performance status (KPS), were obtained from TCGA-GBM Project (see **Supplementary Material E2**).

In this paper, OS was defined as the time between the date of pathological diagnosis and the date of death or the date of last clinical follow-up. PFS was calculated from the date of initial diagnosis to the new tumor event whether it was (a) a progression of disease, (b) local recurrence, (c) distant metastasis, or (d) new primary tumors at all sites or the time the patient was last known to be alive. Similarly, DSS was measured from the date of initial diagnosis until death from the GBM or last follow-up examination. Then, these patients were divided into long-term and short-term survival groups respectively according to the median of OS, PFS, and DSS. Next, all patients were randomly separated into a training cohort (OS and PFS: n = 94; DSS: n = 90) and a validation cohort (OS and PFS: n = 40; DSS: n = 37) at a ratio of 2:1. In this retrospective study, the requirement for informed consent was waived because all the patient data in TCGA were deidentified.

**Figure 1:**
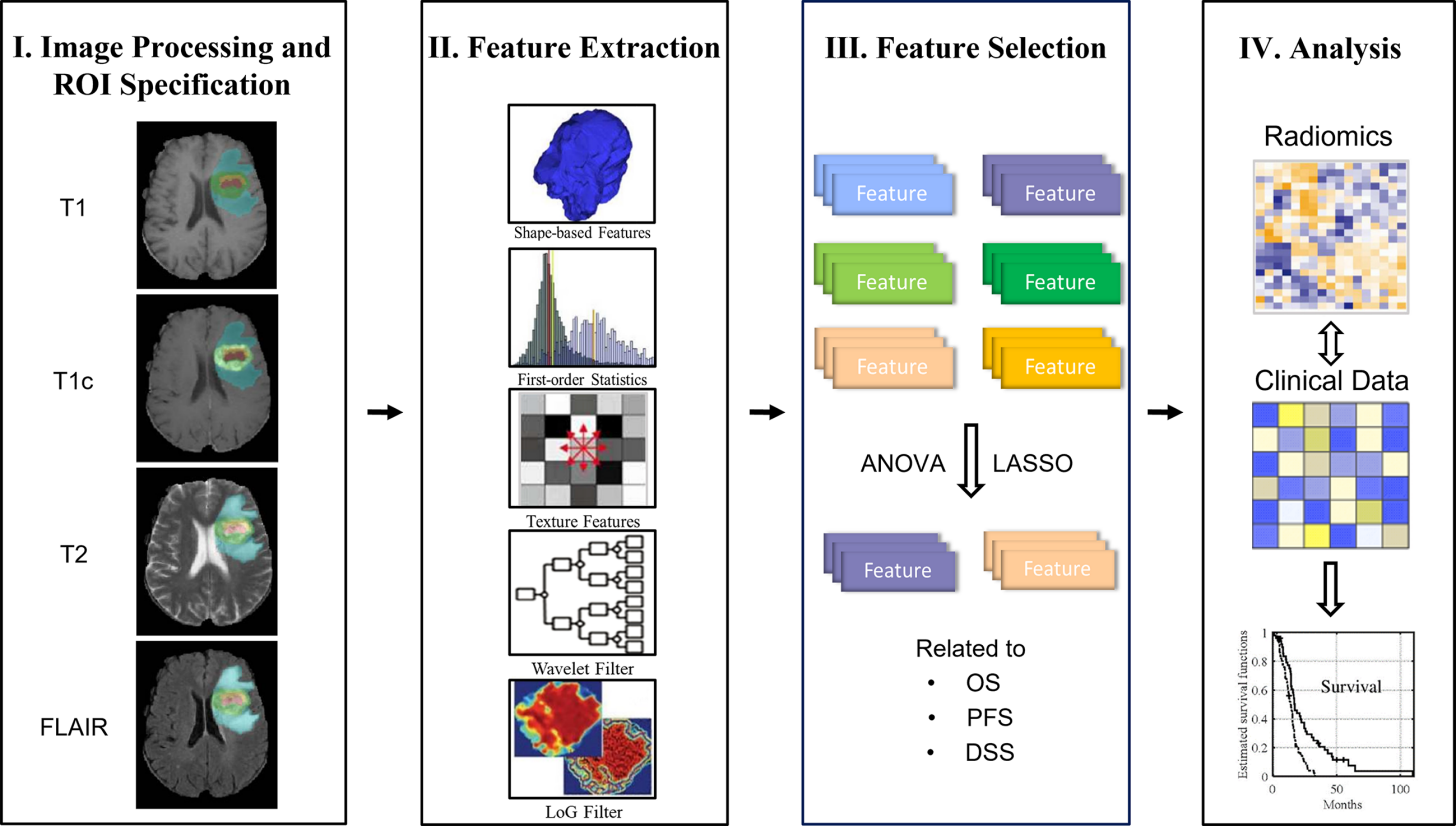
Overall workflow of the study.

The main design idea of this paper is as follows: firstly, the multimodal MRI (multiparameter) data of GBM patients are preprocessed; next, the radiomics features are extracted from different regions of interest (ROIs) (multiregions), and then the analysis of variance (ANOVA) and least absolute shrinkage and selection operator (LASSO) methods are used to analyze and screen the extracted imaging features (i.e., radiomics signature) related to the three kinds of survival endpoints. Finally, combined with clinical risk factors such as age, gender, and KPS, the radiomics nomogram, which is based on a Cox proportional hazards model, was analyzed. The overall workflow of the proposed method is shown in **Figure 1**.

### Imaging Data Acquisition and Preprocessing

All 134 patients underwent four MRI modalities, i.e., T1C, T1, T2, and FLAIR sequence. The range of image acquisition parameters of the four MRI sequences is provided in **Supplementary Material E1**, section S1. The matrix size of all the MRI sequences was 128 × 128. Diverse parameters of different MRI sequences were used during image acquisition, which may have a great influence on three-dimensional (3D) analyses; thus, two-dimensional (2D) preprocessing was performed in this study. First, the four modalities of all subjects are co-registered to the same anatomical template. Next, the planar resolution of each modality was uniformly resampled to 128 × 128. Finally, since the TCGA-GBM database contains multisite data, the scanner model, pixel spacing, slice thickness, and contrast vary within the selected cohort. To consider these differences, all images were resampled to a common voxel resolution of 1 mm^3^, and intensities within each volume were normalized to the [0.1] range.

### Multiregional Labeling

**Figure 2:**
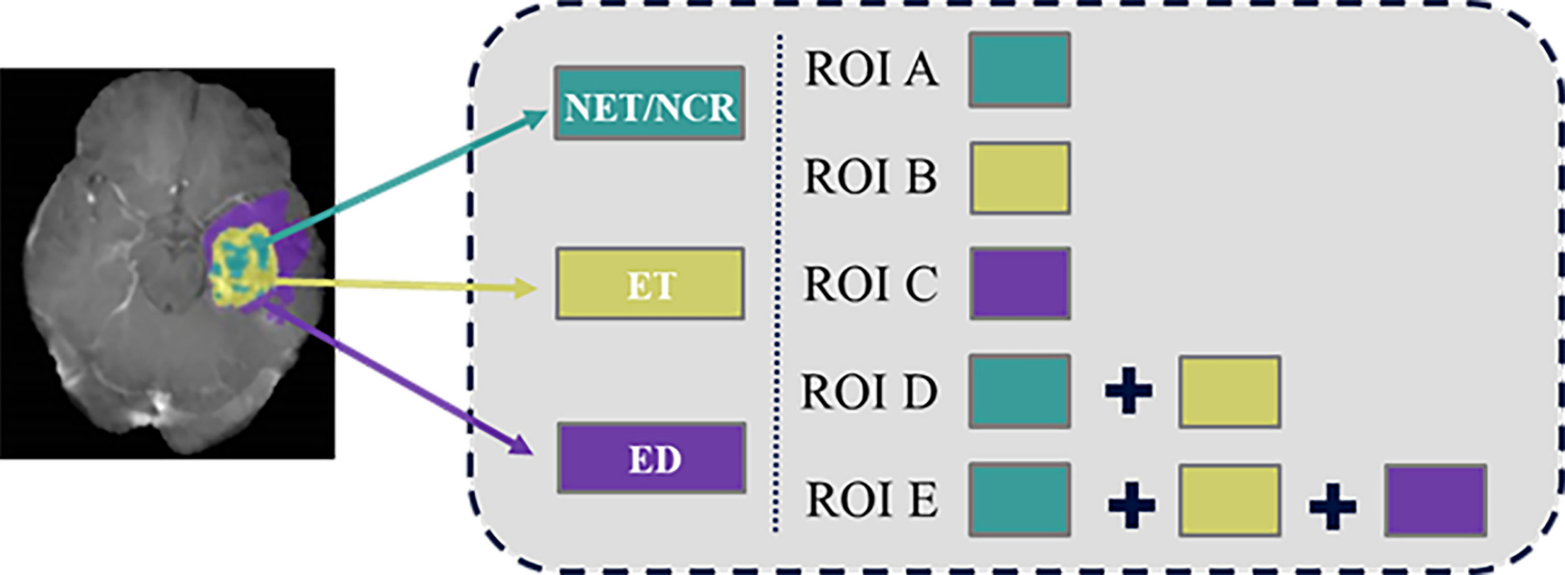
Labeling of the multiple heterogeneous regions.

In order to obtain information about the survival of patients with GBM from multiple tissue types rather than a single tissue type (15), five different heterogeneous regions were drawn (**Figure 2**). Necrosis (NCR) and non-enhancing tumor (NET) region was defined as ROI A, enhancing tumor (ET) core was defined as ROI B, and edema area (ED) was defined as ROI C. Then, ROI D was generated by merging enhancing tumor region and the first ROI. ROI E was generated by adding edema region to ROI D. We also refer to tumor core (TC) as ROI D and whole tumor (WT) as ROI E. Finally, these five regional contours were respectively mapped to each MRI sequence for each patient.

### Radiomics Feature Extraction and Radiomics Signature Construction Related to Survival Indicators

In order to extract high-throughput features, we obtained the original image, eight corresponding Wavelet-filtered images and five corresponding Laplacian of Gaussian (LoG) filtered in each MRI sequence. A total of 5,152 radiomics features were extracted for each patient. A detailed description of the feature extraction is provided in **Supplementary Material E1**, section S2. To reduce the dimension of radiomics features and find out the features that have high evaluation on the prognosis of GBM patients, the ANOVA and the LASSO regression algorithm was adopted to select the survival state-related features among the 5,152 radiomics features in the training cohort.

We use a one-way ANOVA to screen out the characteristics that are separately related to the three survival indicators and have significant differences. Then, LASSO logistic regression method was used to select the characteristics most related to the three survival indicators. LASSO is an effective regression analysis method to constrain the number of independent variables. It can perform feature selection and regularization in high-dimensional data to improve the prediction accuracy through penalty estimation function. The L1 penalty term is added to the ordinary linear model; for ordinary linear model, LASSO estimate is as follows:


$${\hat{\beta }}_{Lasso}=\arg\;\underset{\beta \in R^{d}}{\min}(\|Y-X\beta |{|}^{2}+\lambda \sum_{j=1}^{d}|\beta_{j}|)$$

Where t corresponds to λ one-to-one, which is the adjustment coefficient. If λ is large, it has no effect on the estimated regression parameters, but as λ decreases, the coefficients of most covariates shrink to zero. It makes the model easier to explain: when there are a large number of independent variables, several important independent variables can be found, and the information provided by these variables is the most important in the model.

With the adjustment of λ, the LASSO method can shrink all the coefficients toward zero and set the coefficients of uncorrelated features to zero. Then, 10-fold cross-validation with a maximum area under the curve (AUC) criterion was employed to find an optimal λ, where the final value of λ produces the maximum AUC. The non-zero coefficient is used to construct the regression model, and the corresponding non-zero coefficient is defined as the Rad-score. The fitting formula is generated using a linear combination of selected eigenvalues weighted by their Rad-score. The formula was then used to calculate the radiomics signature of each GBM patient to reflect his or her long-term or short-term survival.


$$\text{Radiomics signature }=\;\sum_{i=1}^{n}\left(p_{i}*v_{i}\right)$$

Where p_i_ (i.e., Rad-score) is the coefficient of the i-th characteristic, and v_i_ is the i-th characteristic value of patients.

### Construction and Assessment of the Radiomics Model With the Training Cohort

The radiomics signature and each clinical factor were first inserted into a Cox proportional hazards model to test whether they were significantly independent prognostic factors for survival stratification in the training cohort. The radiomics signature and significant clinical factors were then utilized to build the Cox proportional hazards model to discriminate the short- and long-term survival outcome of the GBM patients. For comparison, Cox proportional hazards models that used only the significant clinical factors were also established. Finally, based on Cox proportional hazards model, radiomics nomograms of the three survival indicators are respectively constructed, which can directly and individually indicate the probability of survival stratification in the training queue.

The discriminative ability of the radiomics nomogram was quantitatively measured using the C-index, which ranges from 0 to 1. The calibration curves were plotted using observed probabilities and the nomogram-estimated probabilities.

### External Validation of the Radiomics Model on the Validation Cohort

The fitting formula that was constructed with the training cohort was applied to all GBM patients in the validation cohort, and the radiomics signature of each patient was calculated. The radiomics nomogram was then validated in this cohort using the radiomics signatures and clinical factors. Finally, the C-index was implemented to evaluate the model results for survival group stratification. Moreover, the calibration curve and the Kaplan–Meier survival curve were also constructed.

### Clinical Utility of the Radiomics Model

To estimate the clinical utility of the radiomics nomogram, decision curve analysis (DCA) was performed by calculating the net benefits at different threshold probabilities in the combined training and validation cohorts.

### Statistical Analysis

In this study, either Student’s *t* tests or Mann–Whitney *U* tests were applied to confirm whether intergroup differences in continuous variables (such as age) existed between the short- and long-term survival groups. Either chi-square tests or Fisher’s exact tests were performed on the remaining categorical characteristics to determine whether the constituent ratios differed significantly between the groups. All statistical analyses were performed with R software version 3.6.1 (R Foundation for Statistical Computing; http://www.R-project.org, 2019) using basic statistical functions or additional packages. The following R packages were used: the *glmnet* package was used for the LASSO logistic regression, the *rms* package was used for the nomograms and calibration curves, the *Hmisc* package was used for the comparisons between the C-indices, and the *rmda* package was used to implement the DCA.

### Ethics Statement

Ethical review and approval were not required for the study on human participants in accordance with the local legislation and institutional requirements. Written informed consent for participation was not required for this study in accordance with the national legislation and the institutional requirements.

## Results

### Clinical Characteristics of the Patients

The cohort of 40 patients ranged in age from 17 to 84 years with a median OS of 359.5 days, PFS of 195 days, and DSS of 376 days. The clinical characteristics and corresponding results of the statistical analyses comparing the long- and short-term survival groups of OS, PFS, and DSS are summarized in **Table 1**.

**Table 1:** Characteristics of patients in the training and validation cohorts for OS, PFS, and DSS.

		Training cohort		P	Validation cohort		P
		Short-term	Long-term		Short-term	Long-term	
OS	Patient no.	47	47		15	25	
	Age, mean ± SD	62.13 ± 12.72	54.64 ± 12.69	*0.003*	62.53 ± 17.08	51.54 ± 14.36	*0.045*
	Gender, no. (%)			0.052			0.870
	Male	26 (55.3%)	35 (74.5%)		8 (53.3%)	14 (56.0%)	
	Female	21 (44.7%)	12 (25.5%)		7 (46.7%)	11 (44%)	
	KPS, median (1)	80 (40–100)	80 (60–100)	*0.050*	80 (60–100)	80 (60–100)	0.068
	Race, no. (%)			0.536			0.414
	White	40 (85.1%)	42 (89.4%)		11 (73.3%)	21 (84.0%)	
	Others	7 (14.9%)	5 (10.6%)		4 (26.7%)	4 (16.0%)	
	Hemisphere no. (%)			0.778			0.680
	Unilateral	39 (83.0%)	40 (85.1%)		8 (53.3%)	15 (60.0%)	
	Bilateral	8 (17.0%)	7 (14.9%)		7 (46.7%)	10 (40.0%)	
PFS	Patient no.	47	47		17	23	
	Age, mean ± SD	60.74 ± 13.77	56.15 ± 12.35	0.056	59.64 ± 20.34	54.91 ± 11.47	0.234
	Gender, no. (%)			0.668			0.676
	Male	31 (65.9%)	29 (61.7%)		10 (58.8%)	12 (52.2%)	
	Female	16 (34.1%)	18 (38.3%)		7 (41.2%)	11 (47.8%)	
	KPS, median (1)	80 (40–100)	80 (60–100)	*0.008*	80 (60–100)	80 (60–100)	0.171
	Race, no. (%)			0.536			0.201
	White	40 (85.1%)	42 (89.4%)		12 (70.6%)	20 (87.0%)	
	Others	7 (14.9%)	5 (10.6%)		5 (29.4%)	3 (13.0%)	
	Hemisphere no. (%)			0.102			0.622
	Unilateral	38 (80.9%)	31 (65.9%)		15 (88.2%)	19 (82.6%)	
	Bilateral	9 (19.1%)	16 (34.1%)		2 (11.8%)	4 (17.4%)	
DSS	Patient no.	45	45		15	22	
	Age, mean ± SD	60.58 ± 13.70	55.44 ± 12.34	*0.034*	50.13 ± 15.61	52.18 ± 14.58	0.066
	Gender, no. (%)			*0.028*			0.729
	Male	24 (53.3%)	34 (75.6%)		8 (53.3%)	13 (59.1%)	
	Female	21 (46.7%)	11 (24.4%)		7 (46.7%)	9 (40.9%)	
	KPS, median (1)	80 (60–100)	80 (60–100)	0.055	80 (60-100)	80 (60-100)	*0.035*
	Race, no. (%)			0.725			0.890
	White	40 (88.9%)	41 (91.1%)		12 (80.0%)	18 (36.4%)	
	Others	5 (11.1%)	4 (8.9%)		3 (20.0%)	4 (63.6%)	
	Hemisphere no. (%)			0.694			0.417
	Unilateral	41 (91.1%)	42 (93.3%)		9 (60.0%)	16 (72.7%)	
	Bilateral	4 (8.9%)	3 (6.7%)		6 (40.0%)	6 (27.3%)	

P values <0.05 are shown in italics.

DSS, disease-specific survival; KPS, Karnofsky Performance Scale; OS, overall survival; PFS, progression-free survival.

### LASSO Feature Selection and Radiomics Signature Construction

To determine the optimal regulation weight λ for the LASSO algorithm, features with non-zero coefficients for survival stratification were selected by 10-fold cross-validation from the 5,152 radiomics features. The illustration of feature selection using the LASSO algorithm is provided in **Supplementary Material E1**, section S3. A detailed description of the selected non-zero-coefficient features is provided in **Supplementary Material E1**, section S4.

**Figure 3:**
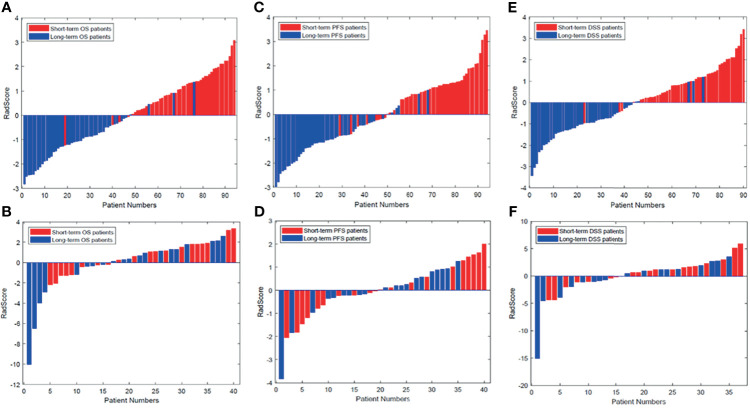
Radiomics signature for each patient in the **(A, C, E)** training cohort and **(B, D, F)** validation cohort. The red bars show the radiomics signature values for the patients with short-term survival (OS, PFS, DFS), and the blue bars show the values for those with long-term survival. DSS, disease-specific survival; OS, overall survival; PFS, progression-free survival.

The radiomics signatures for each GBM patient in the training and validation cohorts are presented in **Figure 3**. The prognostic label represented by the x-axis can clearly distinguish the survival status of GBM patients. That is, the patients with long-term survival generally displayed a significantly higher radiomics signature than the patients with short-term survival in both the training and validation cohorts.

### Non-Zero-Coefficient Features Analysis

**Figure 4:**
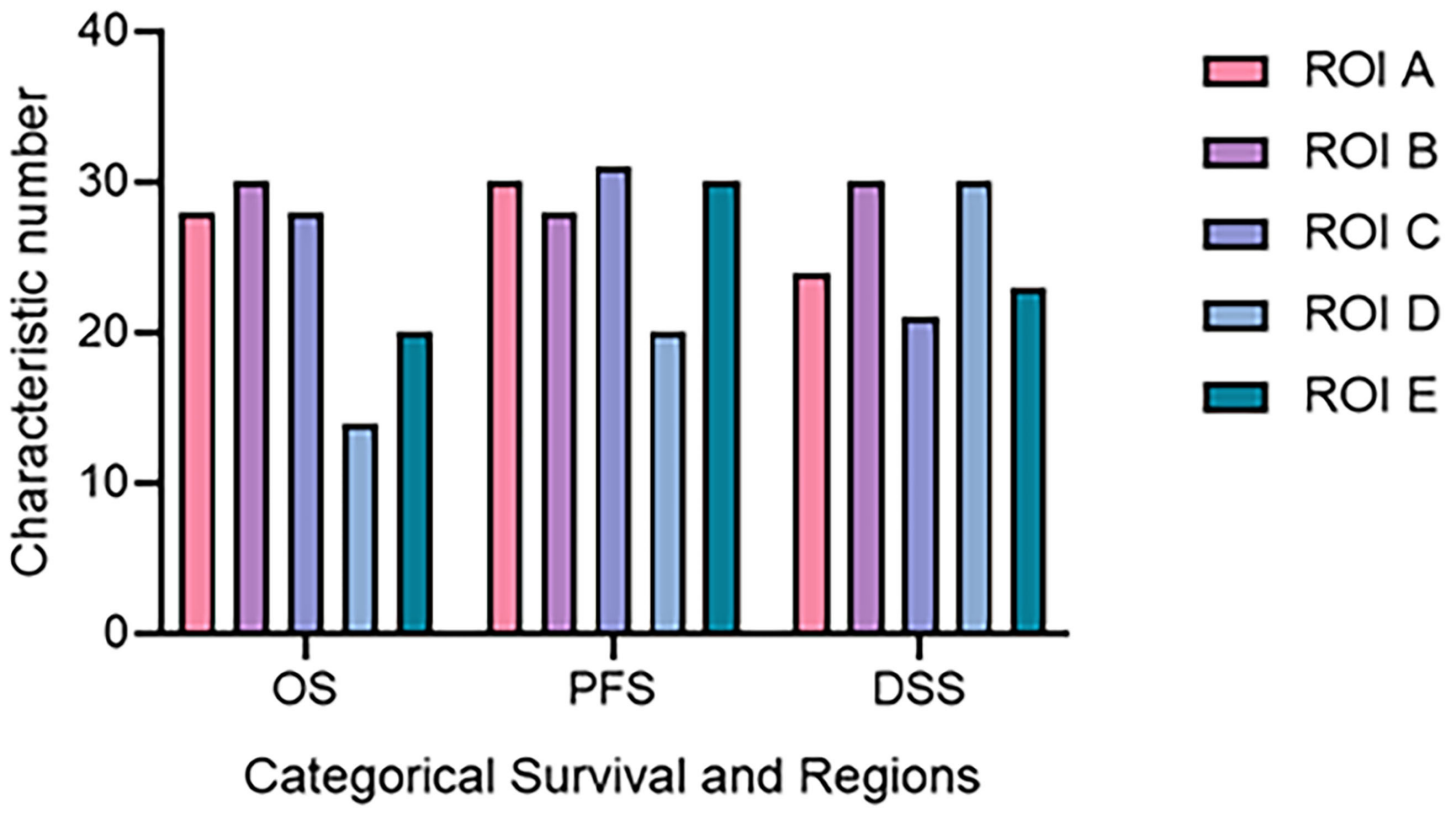
The number of radiomics features corresponding to different ROI and survival time. DSS, disease-specific survival; OS, overall survival; PFS, progression-free survival; ROI, region of interest.

As mentioned above, according to different ROIs and survival endpoints, the radiomics features with non-zero coefficients corresponding to multiregion MRI and three survival periods are selected by LASSO algorithm. The number of different features extracted from each survival indicator and each region is also distinct. According to **Figure 4**, for different survival indicators and different ROIs, the number of non-zero-coefficient radiomics features is inconsistent. As for OS, ROI B has the largest number of features (30 features). For PFS, ROI C has the largest number of features (31 features). Finally, for DSS, ROI A and ROI D have the most features (30 features).

In addition, selected features from different MRI sequences have different coefficients, which can be seen as the importance of one for survival stratification. The results show that T1 sequence and FLAIR sequence account for more than the other sequences. A detailed description of the selected non-zero-coefficient features is provided in **Supplementary Material E1**, section S4.

### The Evaluation of the Radiomics Signature and/or Clinical Risk Factors

The radiomics signature, age, gender, and KPS were identified as independent predictors of survival stratification in GBM patients. The Cox proportional hazards model was applied based on three clinical predictors and/or the radiomics signature. According to **Table 2**, the C-index that resulted from the combined use of the radiomics signature and three clinical predictors for survival stratification was increased significantly for each item in both training cohort and validation cohort.

**Table 2:** The Cox C-index of radiomics signature and clinical factors used for different clinical endpoints of glioblastoma patients in the training cohort and validation cohort.

Cohort	ROI	OS	PFS	DSS
Training	ROI A	0.850 (0.794–0.905)	0.812 (0.756–0.869)	0.845 (0.789–0.901)
	ROI B	0.834 (0.777–0.891)	0.792 (0.731–0.853)	0.844 (0.793–0.894)
	ROI C	0.812 (0.751–0.873)	0.787 (0.751–0.823)	0.784 (0.714–0.855)
	ROI D	0.827 (0.773–0.882)	0.801 (0.744–0.857)	0.851 (0.797–0.904)
	ROI E	0.814 (0.759–0.870)	0.780 (0.722–0.838)	0.828 (0.771–0.885)
	Clinical	0.662 (0.580–0.744)	0.580 (0.489–0.670)	0.631 (0.547–0.715)
Validation	ROI A	0.681 (0.538–0.824)	0.655 (0.516–0.793)	0.724 (0.594–0.854)
	ROI B	0.725 (0.590–0.859)	0.676 (0.540–0.811)	0.710 (0.585–0.836)
	ROI C	0.723 (0.579–0.870)	0.678 (0.540–0.814)	0.710 (0.582–0.838)
	ROI D	0.675 (0.528–0.821)	0.670 (0.533–0.805)	0.700 (0.565–0.833)
	ROI E	0.685 (0.540–0.830)	0.668 (0.528–0.807)	0.706 (0.586–0.825)
	Clinical	0.645 (0.538–0.732)	0.606 (0.527–0.705)	0.611 (0.560–0.692)

The 95% confidence interval is indicated in ().

DSS, disease-specific survival; OS, overall survival; PFS, progression-free survival; ROI, region of interest.

In addition, it can be seen from **Table 2** that tumor subregions have different manifestations in the three survival indicators in the validation set. For different heterogenous regions, the C-index of ROI B was 0.725 (95% CI, 0.590–0.859) for OS, higher than those of the other subregions. However, for PFS, the C-index of ROI C was 0.678 (95% CI, 0.540–0.814), and for DSS, the C-index (0.724, 95% CI, 0.594–0.854) of ROI A is the highest. Furthermore, the results on the whole dataset are shown in **Supplementary Material E1**, Section S7, for a more comprehensive assessment.

### Radiomics Nomogram Construction and Validation

**Figure 5:**
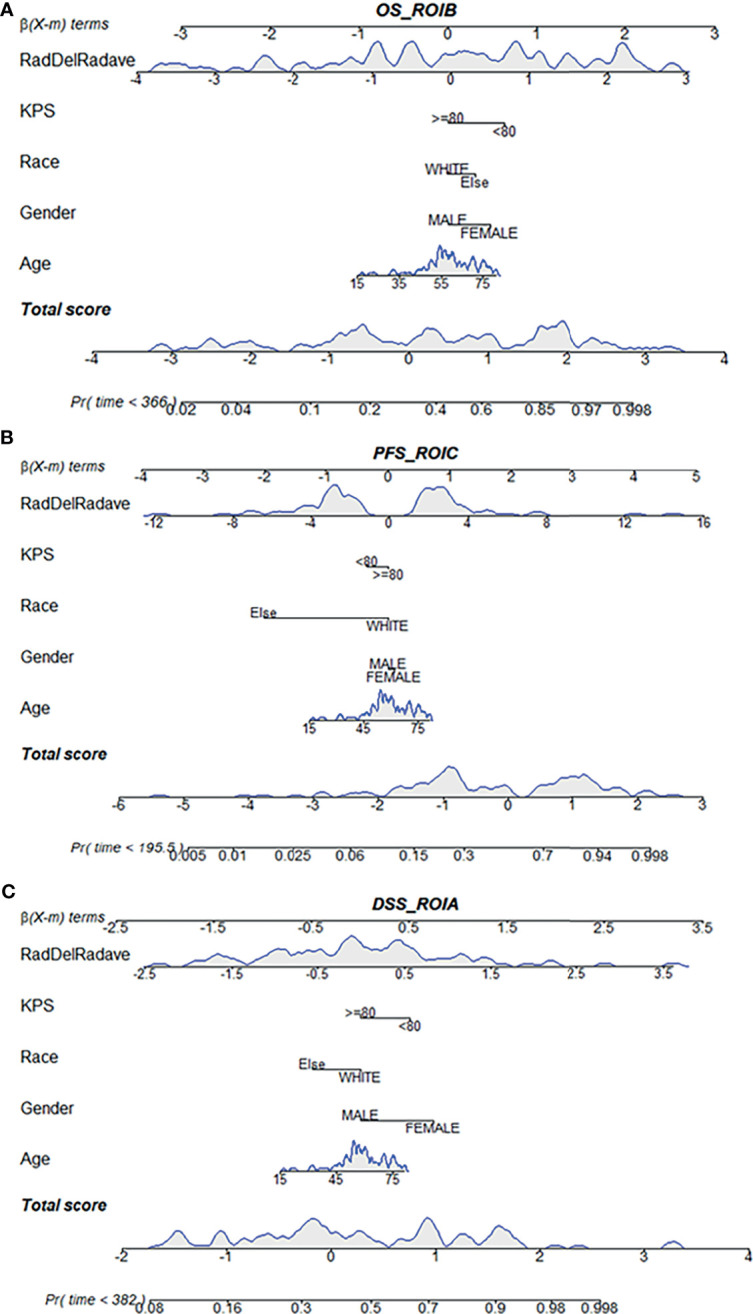
Radiomics nomogram for the three survival indicator stratification of GBM patients. The shaded part indicates the distribution status and probability density of the patients. **(A)** Represents the OS_ROI B model, **(B)** PFS_ROI C model and **(C)** DSS_ROI A model. DSS, disease-specific survival; KPS, Karnofsky Performance Scale; OS, overall survival; PFS, progression-free survival; ROI, region of interest.

**Figure 6:**
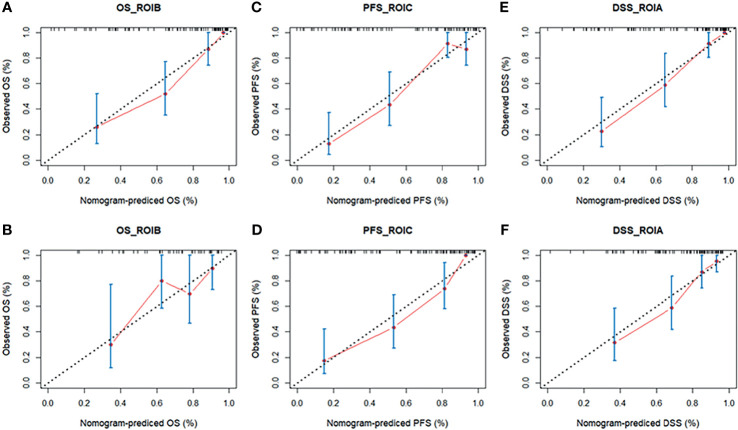
The calibration curves of the radiomics nomogram in the training cohort **(A, C, E)** and validation cohort **(B, D, F)** of the three survival groups. The calibration curves depict the calibration of the nomogram in terms of the agreement between the predicted risk and the observed risk of survival. DSS, disease-specific survival; OS, overall survival; PFS, progression-free survival; ROI, region of interest.

**Figure 7:**
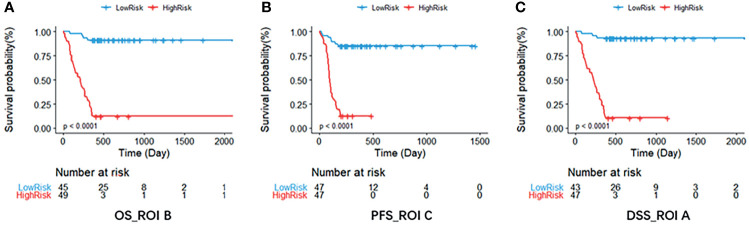
The Kaplan–Meier survival curve shows OS **(A)**, PFS **(B)**, and DSS **(C)** risk stratification for patients with the optimal models in the validation dataset. Patients were classified as low risk and high risk according to radiomics signature. DSS, disease-specific survival; OS, overall survival; PFS, progression-free survival; ROI, region of interest.

Based on the multivariate Cox regression, the radiomics nomogram that incorporated the radiomics signature and the three clinical factors was constructed (**Figure 5**). **Figures 5A–C** respectively represent the prediction models with the highest C-index of OS, PFS, and DSS for GBM patients. **Figure 6** illustrates the calibration curve of the proposed nomogram based on the training cohort. Moreover, favorable calibrations (**Figure 6**) and Kaplan–Meier survival curves (**Figure 7**) confirmed the three optimal models (with the highest C-index) of the validation cohort. The Kaplan–Meier survival curves of the model corresponding to other subregions is shown in the **Supplementary Material E1**, section S5.

### Clinical Utility of the Radiomics Nomogram

**Figure 8:**
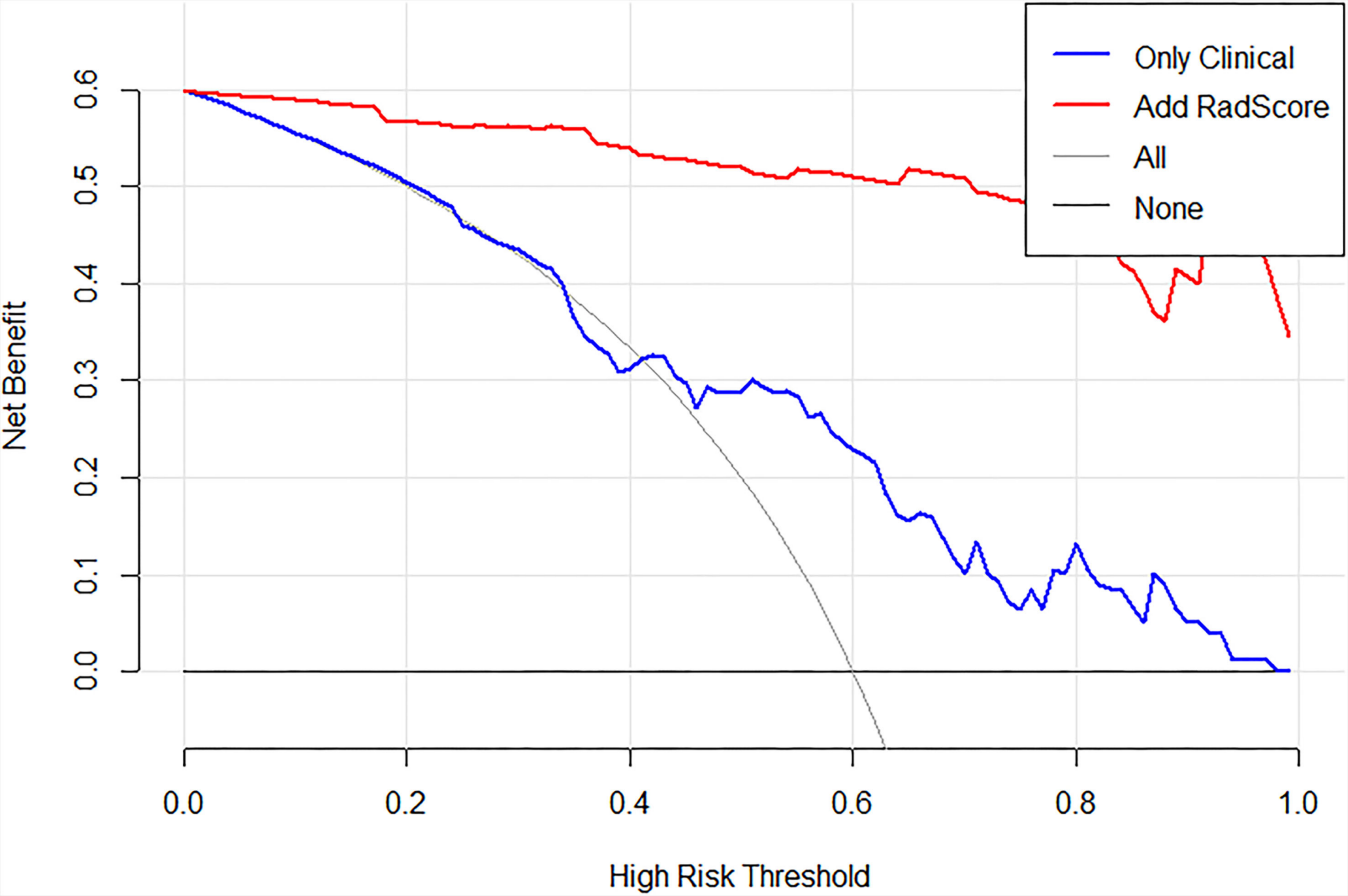
Decision curve analysis (DCA) for the proposed radiomics nomogram, take OS_ROIB as an example. The y-axis represents the net benefit. The x-axis represents the threshold probability. The black line at the bottom represents the hypothesis that no patients had long-term survival. The blue line represents the hypothesis that all patients had long-term survival. The red line represents the net benefit of the radiomics nomogram at different threshold probabilities.

The decision curve for the radiomics nomogram indicates that the use of the radiomics nomogram (**Figure 8**) to stratify the survival of GBM patients was beneficial at all threshold probabilities in our study.

## Discussion

This study investigated the prediction of multiple survival of GBM patients based on multiparametric MRI. The radiomics signature involving multiscale texture features, combined with clinical risk factors, could precisely predict the individualized probability of survival stratification for each GBM patient. The performance differs from GBM patients in OS, PFS, and DSS. Furthermore, different tumor subregions and different modalities of MRI play a significant role in the prognosis of GBM patients.

### The Radiomics Signature Combined With MultiScale Texture Features Increased the Fit and Accuracy of the Model Significantly

In our study, the incremental value when adding the radiomics signature to the clinical factor-based nomogram was assessed. According to the C-index and Decision curves, the combination of the radiomics signature and clinical predictors demonstrated an enhanced stratification efficacy in both the training and validation cohorts of the three survival indicators. The results suggested that the radiomics signature was more robust than the traditional clinical risk factors, in accordance with many previous studies focusing on radiomics nomogram (16–19).

To develop the radiomics signature, more features are added from original and derived images. More features result from the features based on Wavelet transform and LoG transform and have coefficients of higher importance that influenced the radiomics signature model in terms of survival. Previous studies have shown that multiscale texture analyses of MRI based on feature extraction can automatically predict the survival time (OS and PFS) with a precision and speed beyond the scope of human visual analysis (10, 19, 20). For example, texture features based on gray-level co-occurrence matrix (GLCM) and LoG filter extracted from relative cerebral blood volume maps in contrast-enhancing and non-enhancing regions of GBM tumors were found to predict survival outcomes of GBM patients (21). As demonstrated in this study, more radiomic features need to be added to various survival predictions of GBM (19, 22).

### The Distinct Performance for the Three Survival Endpoints of Glioblastoma Patients

In order to evaluate and predict the survival status of GBM patients comprehensively and systematically, we examined three different survival indicators: OS, PFS, and DSS, which are the appropriate clinical endpoints for GBM (4). Among the three outcome indicators in this study, OS is an important and commonly used clinical endpoint, with the advantage that it is convenient to record, because it is not difficult to confirm the date of death and there is minimal ambiguity in defining an OS event (23, 24). However, using OS as an endpoint may weaken a clinical study, as deaths because of non-cancer causes do not necessarily reflect tumor biology, aggressiveness, or responsiveness to therapy. DSS can respond to clinical benefits in a targeted manner, and its enhancement can well reflect the clinical benefits of specific diseases, and the deaths caused by specific diseases are reduced or increased. PFS increased node of “deterioration,” and “deterioration” is often earlier than death, so the follow-up time of PFS is often shorter than OS and DSS. In view of the relatively short clinical follow-up records of some patients, PFS is generally considered to be a better choice of clinical endpoint than OS and DSS (4). The improvement of PFS includes “no deterioration” and “no death,” which indirectly and directly reflect the clinical benefits. Some cancer-related prognostic studies have also shown that OS, PFS, and DSS are important survival indicators, which are closely related to the prediction of clinical benefits (25–29).

Our results showed that the OS and DSS results of GBM patients were relatively consistent, which may be due to the definition of DSS as death caused by a specific disease. And this study focused on glioblastoma multiforme and illustrated that GBM has fewer complications and high mortality. However, in the process of evaluating PFS, it is found that the result of PFS was biased. It is probably due to the large differences between individual patients and the greater changes in imaging characteristics for the progression of tumors. This finding is consistent with another previous study of GBM showing that the stratification of the PFS resulted in worse performance than OS (30).

### Tumor Subregions Have Different Manifestations in the Three Survival Indicators

Our approach is based on comprehensive quantitative information from four different MRI sequences and six heterogeneous regions that enable a multiparametric three-dimensional characterization of the entire tumor. The selected radiomics features were from different heterogeneous regions and sequences of MRI. According to our research, the absolute values of coefficients obtained by the LASSO algorithm indicate the contribution of specific features for survival stratification.

Concerning different heterogeneous regions, the features from the enhancing tumor core (ROI B) contributed more to the OS stratification than did the features from other subregions. Most GBM prognosis-related studies indicated the association between poor prognosis and radiomics features from contrast-enhanced regions (22, 31). For PFS, our results show that ROI C has the highest C-index, which was the edema area. Some recent studies also revealed the role of features from peritumoral brain edema (3, 32). As for DSS, the C-index of region A is the highest, that is, NCR core and NET region. Previous studies have confirmed that the non-enhancement area of GBM patients is associated with their survival (9). Some recent studies also revealed the role of features from central NCR (17, 33). These results further suggested the role of information contained in non-contrast-enhanced subregions and sequences for GBM prognosis. Our study therefore suggests that subregions of GBM may complement disease stratification of patients with GBM and thereby potentially improve clinical.

### Multiparametric MRI Contributed Differently to Predict Survival Stratification of Glioblastoma Patients

In addition, our results show that the selected features based on T1 sequence and FLAIR sequence account for more proportion (see **Supplementary Material E1**, section S4), which is also confirmed in previous studies (30). A comprehensive imaging–genomic analysis of human GBM by using quantitative MRI volumetrics and large-scale genetic and microRNA expression profiles demonstrated the potential for molecular subtyping based on FLAIR (or NER) signal intensity abnormality (34). In another study of high-grade gliomas, Pope et al. (35) analyzed 15 imaging variables obtained from T1-weighted MR images and showed that the presence of non-contrast-enhancing tumor was one of the three variables associated with OS.

In order to more concretely analyze the performance of single-modality and multiparameter MRI, we supplemented the comparison of their C-index of model obtained by each modality on the training set and validation set, respectively (see **Supplementary Material E1**, section S6). The experimental results show that the survival prediction model based on T1 or FLAIR sequence is indeed better than other single modality. However, the performance is not as good as that of multiparameter MRI. When comparing our results with those of older studies, it must be pointed out that radiomics analysis may reveal new insights into the underlying heterogeneity of cancers, driving a valuable prospect to noninvasively delve into GBM heterogeneity (32).

### Several Limitations

Some limitations of this study need to be further investigated. First, the number of enrolled subjects is relatively small in our study, and 134 subjects in TCGA-GBM dataset were used, resulting in low robustness results. This is because we have adopted relatively strict exclusion criteria such as patients with incomplete OS, PFS, and DSS data will be excluded. However, to solve this problem, we use cross-validation in the training process of building the model, which makes our result generalizable to the population. Second, the 2D manual segmentation used in this study may induce bias about tumor slice selection and manual ROI delineation. Furthermore, Hainc et al. (36) have investigated that the variation of slices and ROI delineation method could affect the radiomics features. These findings could be the guidance for our future work. Finally, although with high efficiency and sparsity, LASSO regression method may be less stable when a large number of features were included in the model. Other feature selection methods should be investigated in the future work.

Imaging-related limitations may result from the limited through-plane resolution of the T2 and FLAIR data compared to the higher-resolution T1 data. As a result, the assessment of fine structural details in one of the three spatial dimensions on the FLAIR data was hampered by some degree of blurring.

In conclusion, this study provides reasonable evidence of radiomics based on multiparametric MRI in assessing OS, PFS, and DSS of GBM patients. The features based on diverse regions correlate significantly with GBM survival. Disparate MRI modalities and subregions can provide distinctive but supplemental information. Compared to several survival analysis studies of GBM patients (22, 30, 32), the focus of this study was on the proposed radiomics model, which integrated radiomics signature of heterogeneous regions and three clinical predictors, and can visually and individually estimate the probability of multiple survival stratification for each GBM patient, which suggests its great potential for clinical application. In the future, prognostic research on GBM could consider focusing on the tumor regions mentioned in this paper that have a significant impact on the three survival indicators.

## Data Availability Statement

The original contributions presented in the study are included in the article/**Supplementary Material**. Further inquiries can be directed to the corresponding author.

## Ethics Statement

Ethical review and approval were not required for the study on human participants in accordance with the local legislation and institutional requirements. Written informed consent from the participants’ legal guardian/next of kin was not required to participate in this study in accordance with the national legislation and the institutional requirements.

## Author Contributions

BW and SZ conceptualized and designed the study, developed the methodology, and analyzed and interpreted the data. SZ, YY and XW performed the data collection or acquisition, statistical analysis. LC participated in discussion and language editing.YL, DL and TY reviewed the manuscript. All authors contributed to the interpretation of the results, drafting the manuscript work or revising it critically for important intellectual content and approval of final version to be published and agreement to be accountable for the integrity and accuracy of all aspects of the work.

## Funding

This study was supported by the National Natural Science Foundation of China (62176177, 61873178, 61906130) and the National Key R & D Program of China (2018AAA0102604).

## Conflict of Interest

The authors declare that the research was conducted in the absence of any commercial or financial relationships that could be construed as a potential conflict of interest.

## Publisher’s Note

All claims expressed in this article are solely those of the authors and do not necessarily represent those of their affiliated organizations, or those of the publisher, the editors and the reviewers. Any product that may be evaluated in this article, or claim that may be made by its manufacturer, is not guaranteed or endorsed by the publisher.

## Footnotes

1
cancergenome.nih.gov

2
www.cancerimagingarchive.net/
