# Supplementary material for: Multiple Survival Outcome Prediction of Glioblastoma Patients Based on Multiparametric MRI
Source: Front Oncol. 2021 Nov 25;11:778627. doi: 10.3389/fonc.2021.778627 (PMC8655336; doi:10.3389/fonc.2021.778627)
Supplement: Supplementary file 2 [file DataSheet_1.docx]

Supplementary Material E1

**Section S1.** **The image acquisition parameters of the four MRI sequences**

**Section S2. Detailed description of the radiomics feature extraction**

**Section S3.** **Illustration of feature selection using the LASSO algorithm.**

**Section S4. Features with non-zero coefficients selected by LASSO**

**Section S5. Illustration of Kaplan-Meier survival curve.**

**Section S6.** **Comparison of multiparametric MRI and single modality MRI.**

**Section S7. The performance of a cross-validation on the 134 dataset.**

# Section S1. The Image Acquisition Parameters of the Four MRI Sequences

All 134 patients underwent four MRI modalities, i.e., T1C, T1, T2, and FLAIR sequence. The following table shows the range of image acquisition parameters of the four MRI sequences.

**Table S1.** The range of image acquisition parameters of the four MRI sequences.

| **Sequences** | **repetition time (TR)/echo time (TE)** | **slice thickness** | **spacing slice** |
| --- | --- | --- | --- |
| T1C | 4.9–3285 msec/2.1–20 msec | 1–5 mm | 0.6–7.5 mm |
| T1 | 352–3379 msec/2.75–19 msec | 1–5 mm | 2–7.5 mm |
| T2 | 700–6370 msec/15–120 msec | 1.5–5 mm | 1.5–7.5 mm |
| FLAIR | 6000–11,000 msec/34.6–155 msec | 2.5–5 mm | 2–7.5 mm |

# Section S2. Detailed Description of the Radiomics Feature Extraction

In this study, a total of 5152 candidate radiomics features were generated for each subject (1288 radiomics features × 4 MRI sequences); especially, the more features are added which from original and derived images (1 level of Wavelet decompositions yielding 8 derived images and images derived using LoG filters with 5 sigma levels). The basic features were divided into shape-based features, first-order features, and textural features. All feature extraction methods were implemented using the open-source Python package PyRadiomics(Griethuysen, Fedorov, Parmar, Hosny, & Aerts, 2017).

**Wavelet filter:** The three-dimensional (3D) wavelet filter was used to decompose the original image of each MRI sequence, which can be regarded as preprocessing prior to feature extraction. By changing the ratio of the high-frequency to low-frequency signals in the images, the wavelet transform increases the information from the low-frequency signal. *L* and *H* are a low-pass and high-pass function, respectively, *I* is the original image, and the wavelet decompositions of *I* in 2 directions (x, y, z) are labeled *I_LLL_*, *I_LLH_*, *I_LHL_*, *I_LHH_*, *I_HLL_*, *I_HLH_*, *I_HHL_*, and *I_HHH_*. Then, we can obtain 8 new filtered images. The size of each filtered image is equal to that of the original image and is shift-invariant.

**Log filter:** The Laplacian of Gaussian filter is applied to the input image and a derived image is generated for each Sigma value specified. LoG emphasizes areas of gray level change, where sigma defines how coarse the emphasized texture should be. A low sigma put emphasis on fine textures (change over a short distance), where a high sigma value emphasizes coarse textures (gray level change over a large distance).

**Shape-based Features:** Shaped-based features describe the geometric appearance of the region of interest. In this group of features we have included descriptors of the three-dimensional size and shape of the ROI. These features are independent of the gray level intensity distribution in the ROI and are therefore only calculated on the non-derived image and mask. Unless otherwise specified, features are derived from the approximated shape defined by the triangle mesh. To build this mesh, vertices (points) are first defined as points halfway on an edge between a voxel included in the ROI and one outside the ROI. By connecting these vertices, a mesh of connected triangles is obtained, with each triangle defined by 3 adjacent vertices, which shares each side with exactly one other triangle. This mesh is generated with a marching cubes algorithm. This algorithm moves a 2x2 cube through the mask space. For each position, the corners of the cube are then marked ‘segmented’ (1) or ‘not segmented’ (0). Treating the corners as specific bits in a binary number, a unique cube-index is obtained (0-255). This index is then used to determine which triangles are present in the cube, which are defined in a lookup table.

**First-order Features:** First-order statistics described the distribution of voxel intensities within the image region defined by the mask through commonly used and basic metrics.

**Textural features:** Textural features are visual characteristics that reflect the homogeneity phenomenon of images and the arrangement of the properties that change slowly or periodically on the body surface. Our textural features mainly included gray-level co-occurrence matrix (GLCM), gray-level run-length texture matrix (GLRLM), gray-level size-zone matrix (GLSZM), neighboring-gray-tone-difference-matrix (NGTDM) and gray-level-dependence-matrix (GLDM) features.

# Section S3. Illustration of Feature Selection Using the LASSO Algorithm.

Lasso logistic regression method was used to select the characteristics most related to the three survival indicators. In this study, we performed Lasso on the radiomic features related to OS, PFS and DSS extracted from different heterogeneous subregions, respectively. The 10-fold cross-validation with a maximum area under the curve (AUC) criterion was employed to find an optimal λ, where the final value of λ produces the maximum AUC. Then, with the adjustment of λ, the Lasso method can shrink all the coefficients toward zero and set the coefficients of uncorrelated features to zero. The illustration is provided in Figure S1, Figure S2 and Figure S3.

**
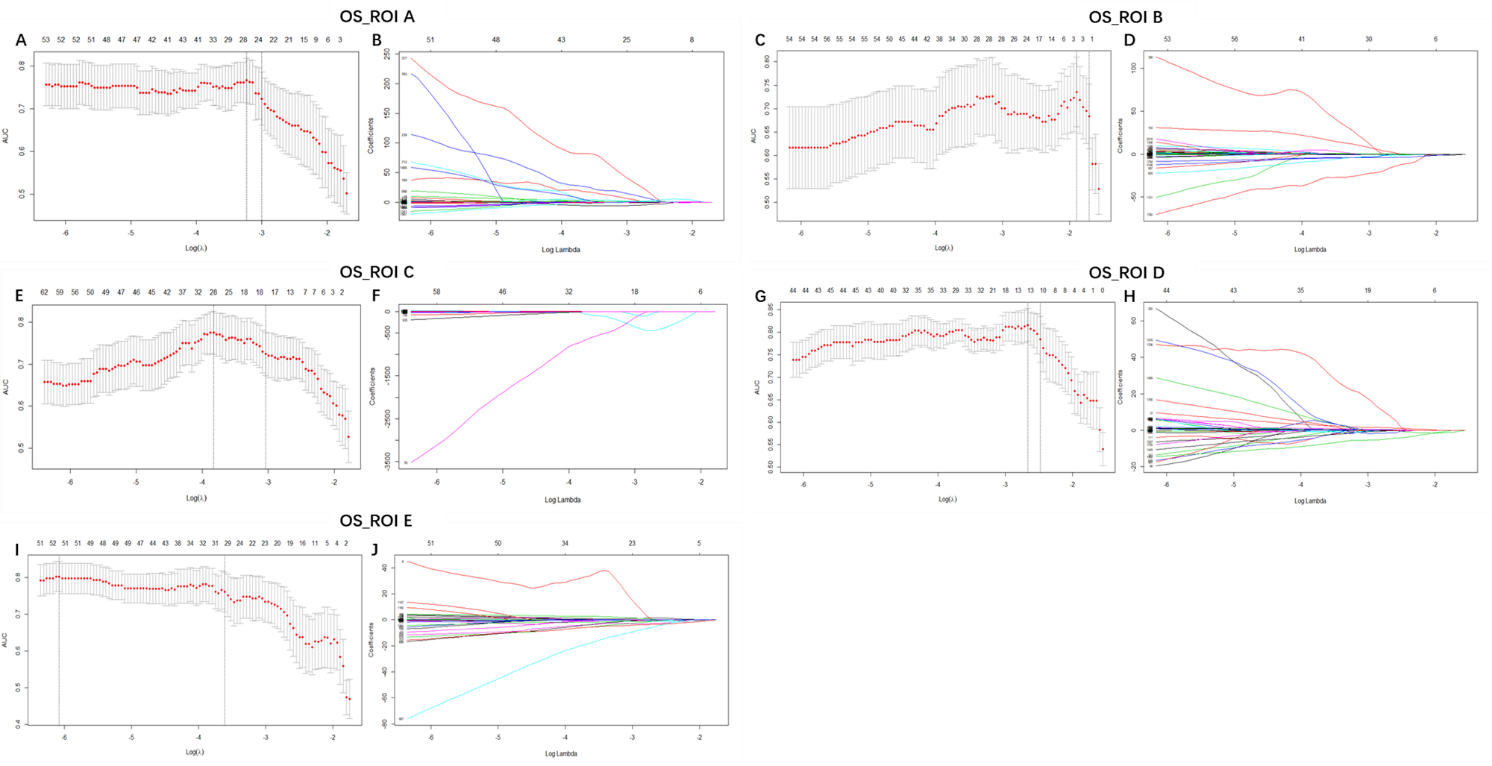
**

**Figure S1.** OS

**
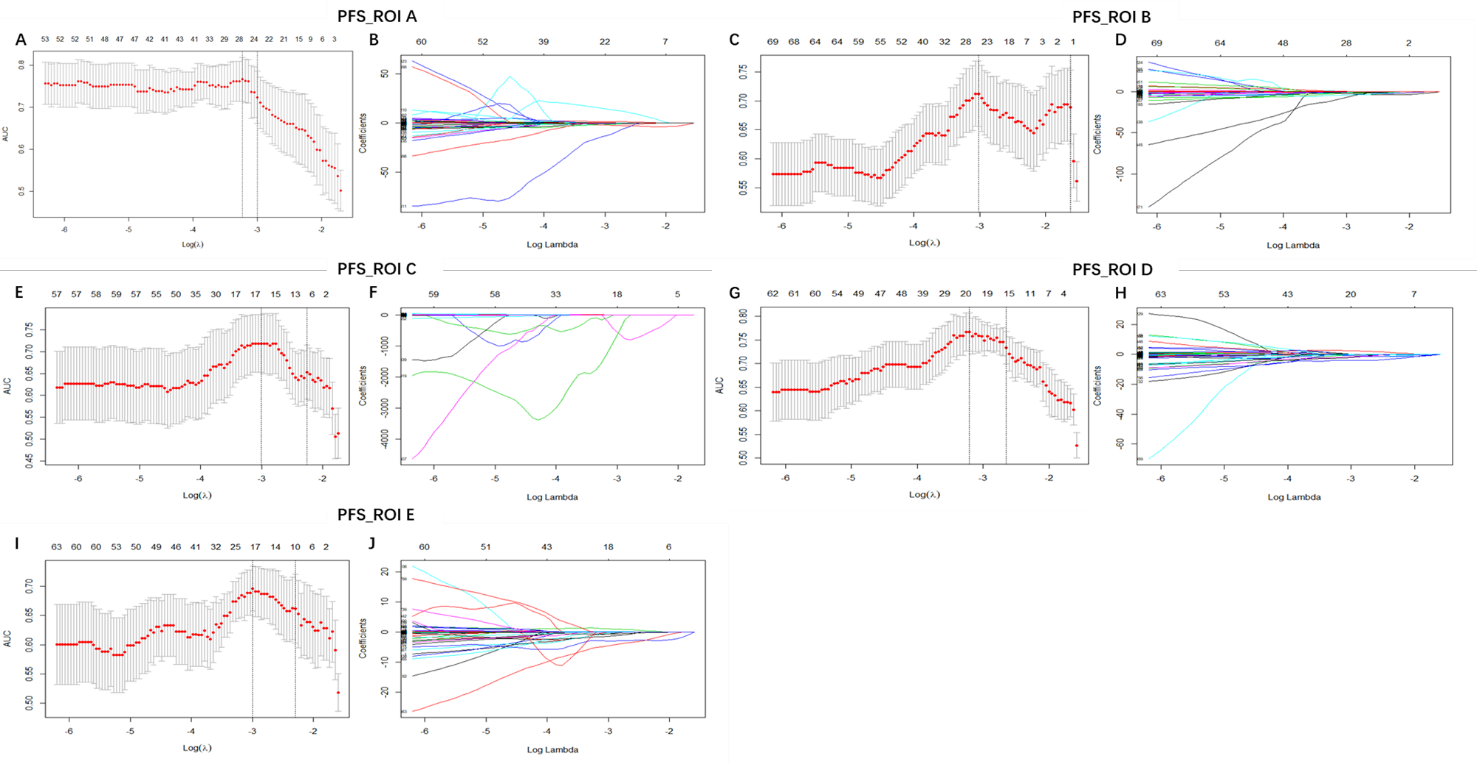
**

**Figure S2.** PFS

**
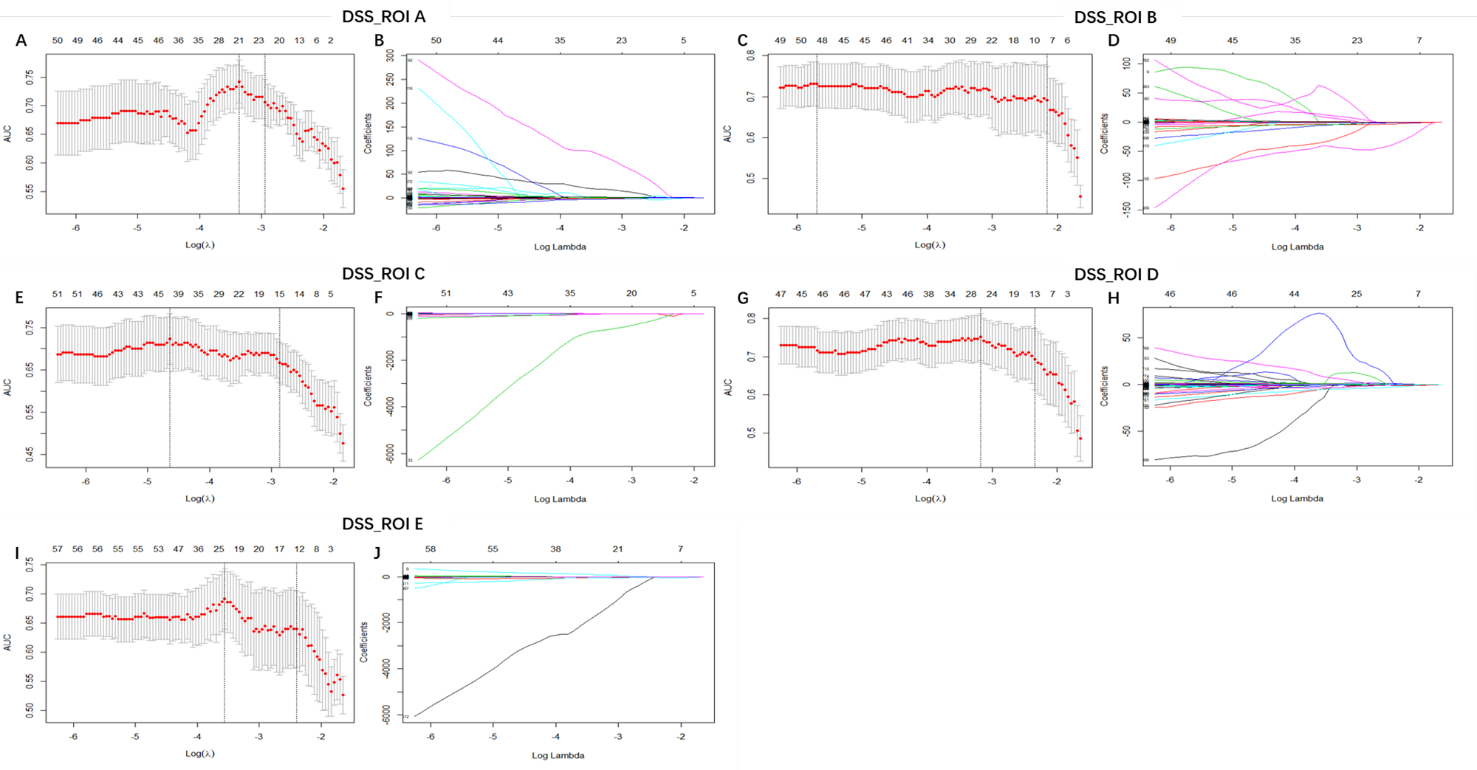
**

**Figure S3.** DSS

**Figure S1, S2 and S3.** Radiomics feature selection using the least absolute shrinkage and selection operator (LASSO) algorithm, Figure S1, Figure S2 and Figure S3 represent features related to OS, PFS and DSS respectively.

In each set of illustrations: (A, C, E, G, I) Selection of the regulation weight λ(Lambda). The AUCs from the LASSO regression cross-validation procedure were plotted as a function of log(λ). The vertical black imaginary lines define the optimal values of λ at which the model provides its best fit to the data. (B, D, F, H, J**)** The LASSO coefficient profiles of the 5152 features. Taking OS-ROI B as an example, the imaginary vertical line was plotted at the selected log(λ) in (A, C, E, G, I), and 30 features with non-zero coefficients were finally identified.

# Section S4. Features with Non-zero Coefficients Selected by LASSO

After feature selection using the one-way ANOVA and the LASSO algorithm, features with non-zero coefficients were selected for the long- and short-term survival stratification. According to the feature extraction process described in **Section S2**, we sorted out the names of these features and their corresponding sequences and coefficients in Table S2. The selected features were ordered by their absolute values of coefficients (which can be seen as the importance of the features).

**Table S2.** The selected non-zero-coefficient features of the three best performing model.

**Table S2(A)** OS_ROI B

|  | **Selected Features** | **Modality** | **Coefficient** |
| --- | --- | --- | --- |
| 1 | wavelet-LLL_glcm_Idmn | T1 | 32.14614971 |
| 2 | log-sigma-4-0-mm-3D_ngtdm_Contrast | FLAIR | -26.55718974 |
| 3 | log-sigma-2-0-mm-3D_gldm_DependenceNonUniformityNormalized | T1 | 12.77775927 |
| 4 | log-sigma-4-0-mm-3D_glszm_SizeZoneNonUniformityNormalized | T1CE | -4.618191327 |
| 5 | wavelet-HHL_glszm_SizeZoneNonUniformityNormalized | FLAIR | -4.179594783 |
| 6 | log-sigma-5-0-mm-3D_glcm_Imc1 | FLAIR | -4.017673183 |
| 7 | wavelet-HHH_glcm_MaximumProbability | FLAIR | 1.552506147 |
| 8 | log-sigma-2-0-mm-3D_firstorder_Skewness | T1 | 0.710613972 |
| 9 | log-sigma-5-0-mm-3D_firstorder_Skewness | FLAIR | 0.689775128 |
| 10 | log-sigma-3-0-mm-3D_glszm_SizeZoneNonUniformityNormalized | T1 | -0.582365859 |
| 11 | wavelet-LLH_firstorder_Median | T1 | 0.42253765 |
| 12 | original_glcm_Imc1 | T1 | 0.370730213 |
| 13 | original_firstorder_Kurtosis | T2 | 0.096456895 |
| 14 | original_firstorder_Kurtosis | T1 | 0.083118011 |
| 15 | wavelet-HLL_firstorder_Median | T2 | 0.07687717 |
| 16 | log-sigma-3-0-mm-3D_gldm_DependenceVariance | FLAIR | 0.057008507 |
| 17 | wavelet-LHL_firstorder_Mean | T2 | 0.033962315 |
| 18 | wavelet-LLH_firstorder_90Percentile | FLAIR | -0.019413601 |
| 19 | wavelet-LHL_firstorder_Median | T2 | 0.018039941 |
| 20 | wavelet-LHL_firstorder_Median | T1CE | 0.006346464 |
| 21 | wavelet-LLL_firstorder_Skewness | T2 | 0.002227181 |
| 22 | wavelet-HLL_glrlm_LongRunHighGrayLevelEmphasis | T1 | -7.59E-05 |
| 23 | log-sigma-5-0-mm-3D_firstorder_RootMeanSquared | T2 | 5.04E-05 |
| 24 | log-sigma-1-0-mm-3D_ngtdm_Busyness | FLAIR | 2.44E-05 |
| 25 | log-sigma-1-0-mm-3D_gldm_GrayLevelNonUniformity | FLAIR | 1.09E-05 |
| 26 | log-sigma-3-0-mm-3D_gldm_GrayLevelNonUniformity | T1 | 5.94E-06 |
| 27 | wavelet-HLL_glszm_LargeAreaHighGrayLevelEmphasis | FLAIR | 2.91E-09 |
| 28 | wavelet-LLL_glszm_ZoneVariance | T1 | 2.88E-09 |
| 29 | original_glszm_ZoneVariance | FLAIR | 2.40E-09 |
| 30 | wavelet-LLH_glszm_LargeAreaEmphasis | FLAIR | 3.65E-11 |

**Table S2(B)** PFS_ROI C

|  | **Selected Features** | **Modality** | **Coefficient** |
| --- | --- | --- | --- |
| 1 | log-sigma-1-0-mm-3D_ngtdm_Coarseness | FlAIR | -2253.178425 |
| 2 | log-sigma-3-0-mm-3D_ngtdm_Coarseness | T2 | -459.283942 |
| 3 | log-sigma-1-0-mm-3D_glcm_Idn | T2 | -14.09235533 |
| 4 | original_glszm_SmallAreaEmphasis | T1CE | -6.327694609 |
| 5 | wavelet-LHL_glrlm_RunPercentage | FlAIR | 4.324511463 |
| 6 | original_glcm_Imc1 | T1 | 3.841959906 |
| 7 | wavelet-LHL_glszm_GrayLevelNonUniformityNormalized | FlAIR | -2.726134223 |
| 8 | wavelet-LLL_ngtdm_Contrast | FlAIR | -2.333920746 |
| 9 | wavelet-HLL_glszm_SizeZoneNonUniformityNormalized | FlAIR | 1.754514087 |
| 10 | original_glcm_Imc1 | T1CE | 1.752238629 |
| 11 | log-sigma-1-0-mm-3D_glcm_Correlation | T2 | -1.618954747 |
| 12 | log-sigma-5-0-mm-3D_ngtdm_Contrast | FlAIR | 1.50772345 |
| 13 | original_ngtdm_Strength | FlAIR | -0.355745186 |
| 14 | log-sigma-1-0-mm-3D_glszm_SmallAreaEmphasis | FlAIR | -0.270773705 |
| 15 | wavelet-LLL_firstorder_Skewness | T1CE | 0.191242657 |
| 16 | wavelet-HLH_firstorder_Skewness | T2 | 0.052412662 |
| 17 | log-sigma-3-0-mm-3D_firstorder_90Percentile | FlAIR | -0.012303487 |
| 18 | wavelet-HHH_firstorder_Skewness | T1 | -0.009803286 |
| 19 | wavelet-LLH_firstorder_90Percentile | FlAIR | -0.007419025 |
| 20 | wavelet-HHL_gldm_LargeDependenceEmphasis | FlAIR | -0.001452188 |
| 21 | log-sigma-5-0-mm-3D_glrlm_LongRunHighGrayLevelEmphasis | T1CE | -0.000760227 |
| 22 | wavelet-HHH_firstorder_Kurtosis | FlAIR | 0.000431238 |
| 23 | log-sigma-5-0-mm-3D_glrlm_LongRunHighGrayLevelEmphasis | FlAIR | -0.000343263 |
| 24 | log-sigma-5-0-mm-3D_ngtdm_Busyness | T1CE | 7.52108E-05 |
| 25 | original_gldm_LargeDependenceHighGrayLevelEmphasis | T1 | -1.22574E-05 |
| 26 | wavelet-LLL_gldm_LargeDependenceHighGrayLevelEmphasis | T1CE | -5.85424E-07 |
| 27 | wavelet-LHH_glszm_LargeAreaLowGrayLevelEmphasis | T1 | 2.1519E-07 |
| 28 | wavelet-LHH_glszm_LargeAreaEmphasis | FlAIR | 3.61716E-08 |
| 29 | log-sigma-3-0-mm-3D_glszm_ZoneVariance | T2 | -1.03398E-08 |
| 30 | wavelet-LHH_glszm_LargeAreaEmphasis | T2 | 5.3476E-09 |
| 31 | original_glszm_LargeAreaHighGrayLevelEmphasis | T2 | 8.77119E-11 |

**Table S2(C)** DSS_ROI A

|  | **Selected Features** | **Modality** | **Coefficient** |
| --- | --- | --- | --- |
| 1 | wavelet-LLL_glcm_Idmn | T1 | 86.07014508 |
| 2 | wavelet-LHH_gldm_SmallDependenceLowGrayLevelEmphasis | T1 | 18.07896186 |
| 3 | wavelet-LLL_ngtdm_Contrast | FLAIR | -2.375514757 |
| 4 | original_glcm_Imc1 | T2 | 1.822503948 |
| 5 | original_glcm_Imc1 | T1 | 1.573258882 |
| 6 | wavelet-HHH_glcm_SumEntropy | T1CE | -0.820166948 |
| 7 | log-sigma-1-0-mm-3D_firstorder_Skewness | T1 | 0.336190027 |
| 8 | original_firstorder_Skewness | T1 | -0.251214316 |
| 9 | wavelet-LLH_firstorder_Mean | T1 | -0.184296645 |
| 10 | original_firstorder_Kurtosis | T1 | 0.115253051 |
| 11 | log-sigma-5-0-mm-3D_firstorder_Kurtosis | T1 | 0.08467036 |
| 12 | wavelet-LLH_firstorder_Median | T1 | -0.047363714 |
| 13 | wavelet-HHH_gldm_SmallDependenceLowGrayLevelEmphasis | T2 | 0.046109463 |
| 14 | wavelet-LHL_glcm_ClusterShade | T1 | 0.036383864 |
| 15 | wavelet-LLH_firstorder_Skewness | FLAIR | -0.027472577 |
| 16 | wavelet-LLH_firstorder_Skewness | T1 | -0.019291364 |
| 17 | wavelet-HHH_firstorder_Skewness | T2 | 0.008239134 |
| 18 | log-sigma-5-0-mm-3D_firstorder_RootMeanSquared | T1 | 0.004834172 |
| 19 | wavelet-HHL_glszm_GrayLevelVariance | FLAIR | -0.004246585 |
| 20 | wavelet-HLH_glszm_GrayLevelVariance | T1 | -0.001483001 |
| 21 | log-sigma-5-0-mm-3D_firstorder_Mean | T1 | 0.000883159 |
| 22 | log-sigma-5-0-mm-3D_glcm_ClusterProminence | FLAIR | -3.65E-06 |
| 23 | log-sigma-1-0-mm-3D_glszm_LargeAreaHighGrayLevelEmphasis | T1 | -8.97E-10 |
| 24 | wavelet-HHH_glszm_LargeAreaHighGrayLevelEmphasis | T1 | -4.11E-10 |

# Section S5. Illustration of Kaplan-Meier Survival Curve.

The Kaplan-Meier survival curves show the risk stratification of different survival indicators in different subregions of patients in the validation dataset. Patients were classified as low risk and high risk according to radiomics signature in all the models we constructed. The illustration is provided in Figure S4.


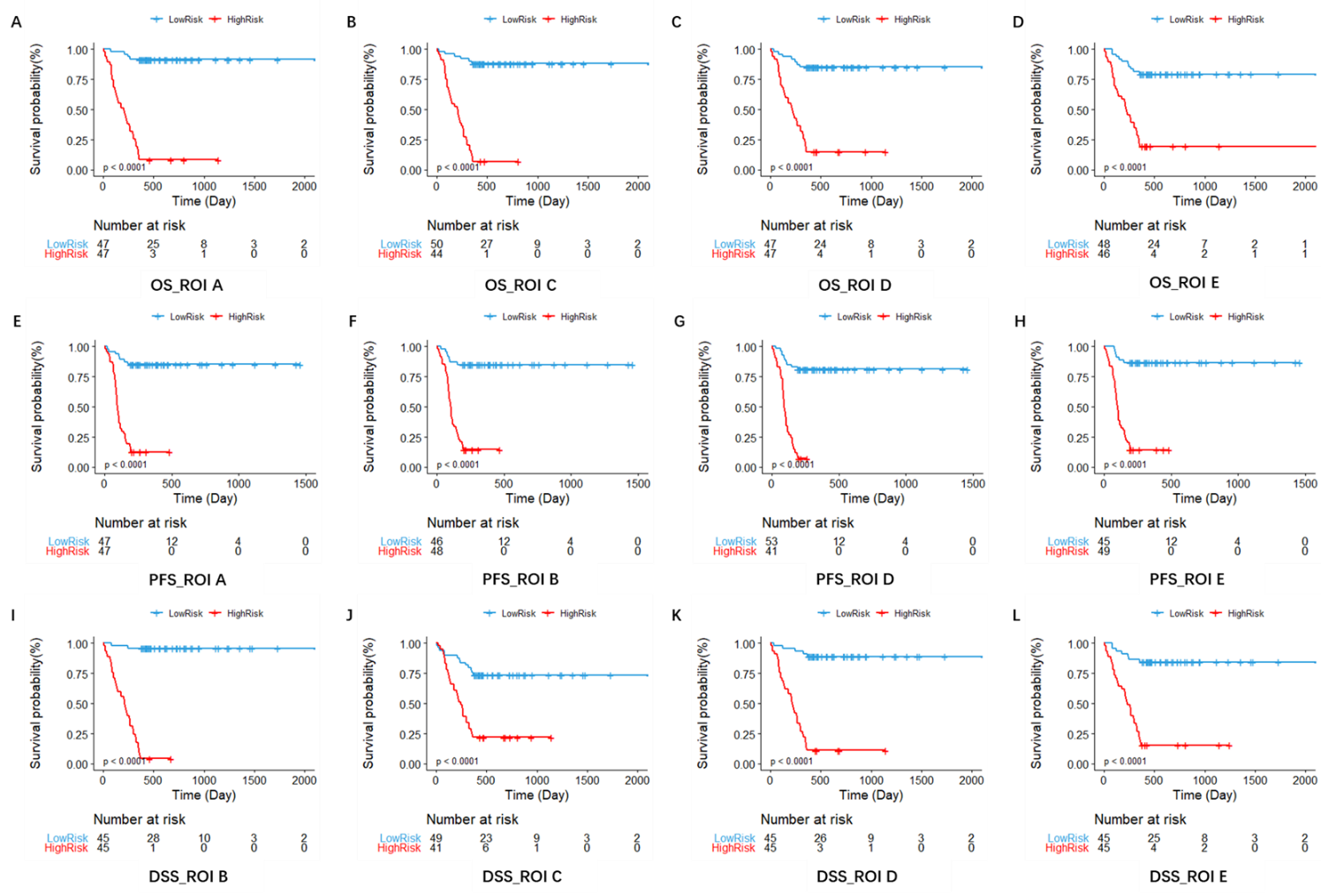


**Figure S4.** Illustration of Kaplan-Meier survival curves of different subregions and different survival indicators.

# Section S6. Comparison of Multiparametric MRI and Single MRI Modality.

To better demonstrate the advantages of our multiparametric MRI, we added the experiments of using only each MRI modality and the comparison with multiparameter MRI. Here take the best three results (OS_ROI B, PFS_ROI C, DSS_ROI A) as an example. The specific results of C-index obtained by multiparameter MRI and single-modality MRI on the validation set were shown in the Table S3 as follows.

**Table S3.** Comparison of C-index obtained by multiparameter MRI and single-modality MRI on the validation set.

|  | Train | | |  | Validation | | |
| --- | --- | --- | --- | --- | --- | --- | --- |
|  | OS_ROI B | PFS_ROI C | DSS_ROI A |  | OS_ROI B | PFS_ROI C | DSS_ROI A |
| T1 | 0.802 (0.744-0.861) | 0.695 (0.626-0.723) | 0.830 (0.774-0.885) |  | 0.706 (0.567-0.845) | 0.670 (0.528-0.812) | 0.718 (0.590-0.848) |
| T1CE | 0.762 (0.698-0.828) | 0.723 (0.656-0.791) | 0.740 (0.664-0.816) |  | 0.702 (0.556-0.847) | 0.677 (0.537-0.818) | 0.701 (0.557-0.845) |
| T2 | 0.764 (0.701-0.828) | 0.692 (0.621-0.763) | 0.742 (0.662-0.822) |  | 0.706 (0.568-0.844) | 0.693 (0.556-0.829) | 0.733 (0.602-0.864) |
| FLAIR | 0.794 (0.727-0.860) | 0.696 (0.622-0.770) | 0.803 (0.741-0.864) |  | 0.710 (0.588-0.832) | 0.691 (0.549-0.832) | 0.719 (0.593-0.846) |
| Multi-modality | 0.834 (0.777-0.891) | 0.787 (0.751-0.823) | 0.845 (0.789-0.901) |  | 0.725 (0.590-0.859) | 0.678 (0.540-0.814) | 0.724 (0.594-0.854) |

The 95% confidence interval is indicated in ().

The results in the table show that the survival prediction model based on T1 or FLAIR sequences outperforms other single modalities. Moreover, the performance of single-modality MRI model is worse than those of multi-parameter MRI.

# Section S7. The Performance of a Cross-validation on the 134 Dataset.

The specific results are shown in the Table S4 below.

**Table S4.** The Performance of a Cross-validation on the 134 Dataset.

| ROI | OS | PFS | DSS |
| --- | --- | --- | --- |
| ROI A | 0.854(0.809-0.901) | 0.824(0.778-0.870) | 0.837(0.787-0.890) |
| ROI B | 0.817(0.771-0.863) | 0.796(0.744-0.848) | 0.853(0.804-0.902) |
| ROI C | 0.809(0.756-0.862) | 0.760(0.702-0.818) | 0.804(0.751-0.857) |
| ROI D | 0.833(0.786-0.811) | 0.781(0.725-0.837) | 0.859(0.816-0.923) |
| ROI E | 0.821(0.771-0.871) | 0.792(0.727-0.857) | 0.824(0.773-0.875) |
| Clinical | 0.666(0.595-0.736) | 0.591(0.518-0.663) | 0.642(0.571-0.712) |

The 95% confidence interval is indicated in ().

Reference

Griethuysen, J., Fedorov, A., Parmar, C., Hosny, A., & Aerts, H. (2017). Computational Radiomics System to Decode the Radiographic Phenotype. *Cancer Research, 77*(21), e104-e107.
